# Supplementary material for: Emergence of Quantum Critical Behavior in Metallic Quantum-Well States of Strongly Correlated Oxides
Source: Sci Rep. 2017 Nov 30;7:16621. doi: 10.1038/s41598-017-16666-x (PMC5709408; doi:10.1038/s41598-017-16666-x)
Supplement: Supplementary file 1 — Supplementary Information [file 41598_2017_16666_MOESM1_ESM.pdf]

**Supplementary Information**

# Emergence of Quantum Critical Behavior in Metallic Quantum-Well States of Strongly Correlated Oxides

Masaki Kobayashi[1,*], Kohei Yoshimatsu[1,2], Taichi Mitsuhashi[1,3], Miho Kitamura[1], Enju Sakai[1], Ryu Yukawa[1], Makoto Minohara[1], Atsushi Fujimori[2], Koji Horiba[1], and Hiroshi Kumigashira[1,3,*]

[1]*Photon Factory, Institute of Materials Structure Science, High Energy Accelerator Research Organization (KEK), 1-1 Oho, Tsukuba 305-0801, Japan*

[2]*Department of Physics, University of Tokyo, 7-3-1 Hongo, Bunkyo-ku, Tokyo 113-0033, Japan*

[3]*Department of Physics, Tohoku University, Sendai 980-8577, Japan*

(Dated: 17th September 2017)

## Sample growth and characterization

Digitally controlled $SrVO_3$ (SVO) ultrathin films were grown onto atomically flat $TiO_2$-terminated 0.05-wt% Nb-doped $SrTiO_3$ (Nb:STO) (001) substrates in a laser molecular-beam epitaxy chamber connected to a photoemission system at a beam line of the Photon Factory, KEK[16–19]. During deposition, the substrate temperature was maintained at 900°C under a high vacuum of $10^{-8}$ Torr. During growth of the SVO film, the film thickness was controlled on the atomic scale by monitoring the intensity oscillation of a specular spot observed in reflection high-energy electron diffraction (RHEED). The period of oscillation corresponded to the deposition of one monolayer (ML) of SVO, which was also confirmed using the deposition rate estimated from grazing-incidence X-ray reflectivity measurements.

The surface morphology of the measured SVO ultrathin films was confirmed by atomic force microscopy (AFM), which showed atomically flat step-and-terrace structures. The crystal structures were characterized using four-circle x-ray diffraction (XRD) and cross-sectional transmission electron microscopy (TEM), which confirmed coherent growth on the substrate and the formation of a chemically abrupt interface between SVO and Nb:STO. Furthermore, these crystallographic characterizations indicated that there were no detectable structural disorders in the prepared SVO ultrathin films grown on Nb:STO(001) substrates. The in-plane lattice constant was $a$ = 3.905 Å, which is identical to that of Nb:STO, whereas the out-of-plane lattice constant was $c$ = 3.82 Å. These characterization results indicated that SVO films could be coherently grown onto Nb:STO substrates with an atomically flat surface and a chemically abrupt SVO/Nb:STO interface, each of which is a precondition for quantum confinement[16–19]. Further details of the growth conditions and characterization of the SVO ultrathin film are described elsewhere[16,17,19]. Low-energy electron-diffraction (LEED) patterns showed sharp 1×1 spots with some superstructure spots of $\sqrt{2}\times\sqrt{2}$-R45° for all samples. The prepared films were transferred under an ultrahigh vacuum of $10^{-10}$ Torr to the photoemission chamber. The in-vacuum transfer was necessary to avoid degradation of the SVO surface upon exposure to air.

The preconditions for the thickness-dependent study of SVO quantum well (QW) states are atomically flat surfaces, chemically abrupt interfaces, and digital control of the layer thicknesses. The films used in the present study have the same qualities as the films used in previous studies[16–19] because we grew the films under the same growth conditions and in the same growth chamber. Homogeneously coherent films with atomically flat surfaces and chemically abrupt interfaces could be obtained in a reproducible manner. In fact, the detailed

characterization provided experimental evidence that the preconditions listed above were fulfilled. The reproducibility of the sample quality was further confirmed by the fact that the subband structures obtained in the present study were almost identical to those of the previously obtained samples, guaranteeing QW structures of the same quality as those used in previous studies[16–19].

## Subtraction of the momentum-independent background from the ARPES spectra

In all QW structures measured, we observed a momentum-independent background contributing spectral weight up to the Fermi level ($E_F$), as shown in Fig. S1. Such backgrounds have been commonly observed in angle-resolved photoemission spectroscopy (ARPES) spectra[8]. Here, such backgrounds may result from angle-integrated spectral weight due to imperfections of the film surface, as well as the broad tail of intensity of the V-3*d* incoherent states located near 1.5 eV. We subtracted the backgrounds according to the procedure of previous ARPES data analyses[8] and applied the subtraction procedure to the data shown in Figs. 1a, 1b, and 2a in the main text. The momentum-independent background was determined by integrating the energy distribution curves (EDCs) outside the Fermi surface (FS), namely in the range $0.45 < k_x < 0.6$ Å$^{-1}$, as shown in Figs. S1a and S1b. The resultant momentum-independent backgrounds for the respective QW structures are shown in Fig. S1c, together with the ARPES spectra inside the FS. The subtraction procedure for the background enables extraction of the intrinsic spectral function relevant to our study and consequently enables precise estimation of the evolution of the ARPES spectral weight as a function of the layer thicknesses. The result of applying this procedure to all of the ARPES images shown in

the main text is summarized in Fig. S2. As can be seen in Fig. S2, the subtraction of background makes the subband structures clear, although all dispersions also appear in the raw data. Note that the background subtraction procedure merely makes these subband structures easier to visualize and does not affect the subband structures themselves.

## Spectral weight transfer from the coherent part to the incoherent part

Figure S3 shows the raw ARPES spectra at the Fermi momentum ($k_F$) in a wider energy range for SVO-QW structures. As can be seen in Fig. S3, it is evident that the spectral weight of the quasiparticle peak close to $E_F$ (the coherent part) is transferred to the lower Hubbard band (the incoherent part) at about 1.5 eV as in the case of the previous AIPES study[17]. As a result of the spectral weight transfer, there is no spectral weight in near-$E_F$ region for a Mott insulating 2-ML film.

## Determination of the self-energy from the ARPES spectra

In order to shed light on the underlying physics, we have evaluated the self-energy $\Sigma(\boldsymbol{k},\omega)$ by employing line-shape analysis of the momentum distribution curves (MDCs) as a function of the binding energy $\omega$[26]. Note that the MDC width $\Delta k$ reflects the imaginary part of the self-energy, $\mathrm{Im}\Sigma(\boldsymbol{k},\omega)$, which is related to the coherent length $l$ of electrons, as follows:

$$\hbar v_k \Delta k = \frac{\hbar v_k}{l} \approx |2\mathrm{Im}\Sigma(\boldsymbol{k},\omega)|. \quad \text{(S1)}$$

Here, $\hbar$ is the Planck constant and $v_k$ is the velocity along the band dispersion. Hence, $\mathrm{Im}\Sigma(\boldsymbol{k},\omega)$ could be deduced from the measured $\Delta k$ values. In a previous ARPES study on SVO-QW structures[18] as well as bulk SVO[19], $\Sigma(\boldsymbol{k},\omega)$ in SVO is particularly independent of $\boldsymbol{k}$.

Thus, if we neglect the $\boldsymbol{k}$-dependence of $\mathrm{Im}\Sigma(\boldsymbol{k},\omega)$ for the observed subbands, $\mathrm{Im}\Sigma(\omega)$ near $E_F$ is given by:

$$|2Z\mathrm{Im}\Sigma(\omega)| \approx \hbar v_F^* \Delta k, \qquad \text{(S2)}$$

where $Z$ is the renormalization factor and $v_F^* = \frac{1}{\hbar}\left(\frac{d\varepsilon(\boldsymbol{k})}{dk_\parallel}\right)$ is the experimentally determined Fermi velocity. Throughout the analysis in this study, the energy ranges for the application of Eq. (S2) were defined so as to maintain the linearity of each $n = 1$ subband from $E_F$[18].

## Absence of detectable kink structures in the ARPES spectra

A kink structure is expected to be observed in the ARPES spectra of the SVO-QW structures in the Fermi liquid regime (SVO-QW structures with layer thickness of 6–8 ML). In the case of bulk SVO (Ref. 19), a weak but distinct kink structure has been observed at ~60 meV, although the kink structure is relatively very weak in comparison with that in cuprates[26]. However, as can be seen in Fig. 3a, it seems to be difficult to identify a kink structure in the ARPES images of the SVO-QW structures. In order to check the existence of the possible kink structure in the SVO-QW structures, we have shown $\mathrm{Im}\Sigma(\omega)$ for 6–8 ML in the Fig. S4. As can be seen in Fig. S4, it is hard to identify a kink structure. It is also confirmed by the fact that there is no detectable deviation between experimental subband dispersion and band structure calculation[16], which corresponds to the absence of characteristic structure in the real part of self-energy $\mathrm{Re}\Sigma(\omega)$.

According to Ref. 19, the kink in bulk $SrVO_3$ is likely due to a coupling of electrons with some phonon modes characteristic to the perovskite oxides. Since SVO is a good conductive

oxide, the weaker kink structure may originate from the strong screening of the electron-phonon coupling by a large amount of conduction carriers[27]. Therefore, it is reasonable to conclude that the kink structures may exist in the Fermi liquid regime of SVO-QW structures but the characteristic structure is buried in the experimental error owing to its weak structure due to strong metallic screening in SVO and a significant contribution from other higher quantized states ($n$ = 2 and 3).

## Estimation of the Fermi-liquid cutoff energy

Another phenomenological form for describing the crossover from a Fermi liquid to non-Fermi liquid has been proposed from a recent ARPES study in cuprates[29]. In order to test the applicability of the form, we have analyzed the experimental self-energy Im$\Sigma(\omega)$ by the same formula as used in the previous ARPES study[29]. According to the previous ARPES study[29], there is a cutoff energy for discriminating between Fermi liquid and non-Fermi liquid excitations. Taking into account for the cutoff energy $\omega_c$, we have tried to reproduce the experimental Im$\Sigma(\omega)$ by the following form:

$$|Z\mathrm{Im}\Sigma(\omega)| = \begin{cases} \Gamma^{\mathrm{imp}} + \beta\omega^2, \omega < \omega_c \\ \gamma + \lambda\omega, \omega > \omega_c \end{cases}, \tag{S3}$$

where $\Gamma^{\mathrm{imp}}$ is the inverse lifetime, $\beta$ is the coefficient for $\omega^2$ term reflecting the strength of the electron correlation, $\gamma$ is the offset at the cutoff, and $\lambda$ is the coefficient for $\omega$-linear term. Note that parameters of $\Gamma^{\mathrm{imp}}$ and $\beta$ are the same as those in the Eq. (1) in the text. Figure S5 is the result of fitting based on the Eq. (S3). As can be seen in Fig. S5a, the fitting using the Eq. (S3) also well reproduces the experimentally obtained Im$\Sigma(\omega)$. The plot of the estimated $\omega_c$ against

layer thicknesses is shown in Fig. S5b. The $\omega_c$ decreases with decreasing layer thickness for the SVO-QW structures. The $\omega_c$ may reach 0 at 3 ML because the data can be well reproduced by $\omega$-linear term only, as shown in Fig. 3a. Meanwhile, the $\omega_c$ for SVO-QW structures above 6 ML are not definitive because the data can be well reproduced by $\omega^2$ term only as shown in Fig. 3a and Fig. S4. The formulae of Eq. (1) and Eq. (S3) are one of the approximate expressions for phenomenologically describing the observed MDC linewidth. The both formulae well reproduce the experimental results within the experimental error bar, indicating the existence of the crossover from a Fermi liquid to a non-Fermi liquid in the SVO-QW structures and the emergency of the quantum critical point in the two-dimensional limit of the metallic QW states.

## Strongly correlated nature of the QW states

In general, the coefficient $\beta$ reflects the strength of the electron correlation[18] and may be negatively correlated with the quasiparticle (QP) weight shown in Fig. 2c. In Fig. S5c, the coefficient $\beta$ is plotted as a function of layer thickness of SVO. As expected from the monotonic decrease of the QP weight (see Fig. 2c), the coefficient $\beta$ monotonically increases with decreasing the layer thickness. These analytical results provide further support for the strongly correlated nature of the QW states.

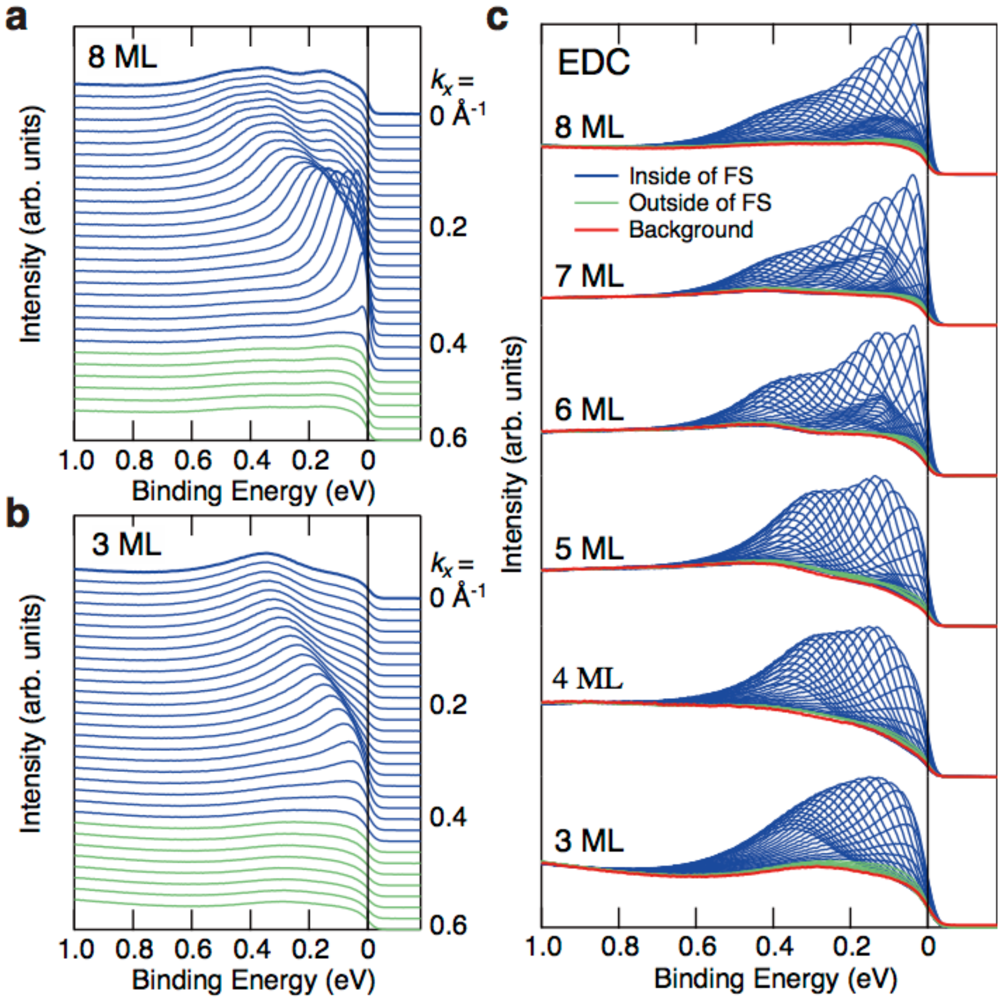


**Figure S1. Raw EDCs in respective SVO-QW structures.** (**a**), (**b**) Raw EDCs of the 8-ML and 3-ML samples, respectively, which are taken along a $k_x$ slice near the X point ($k_y = 0.75\pi/a$) (see a dashed line in the inset of Fig. 1 in the main text). The blue and green curves are the EDCs in the inside of FS and the outside of FS, respectively. (**c**) Momentum-independent backgrounds for the ARPES spectra (red curves), together with the EDCs in the inside of FS (blue) and outside of FS (green). The momentum-independent background of each sample has been determined by integrating its EDCs between $0.45 < k_x < 0.6$ Å$^{-1}$, which is outside FS.

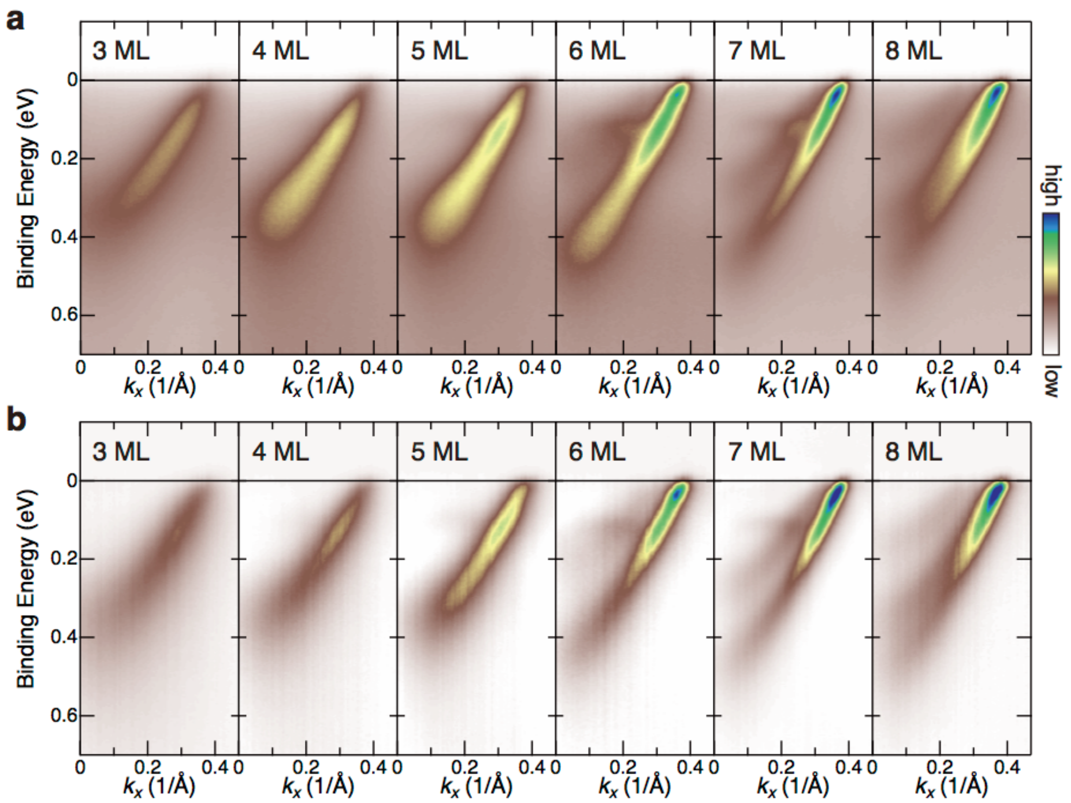


**Figure S2. ARPES images of respective SVO-QW structures before and after subtraction of the momentum-independent background.** (**a**) Raw ARPES images for the 3-, 4-, 5-, 6-, 7-, and 8-ML samples. (**b**) Equivalent ARPES images after subtracting the momentum-independent backgrounds, which are determined by the analytical procedure shown in Fig. S1.

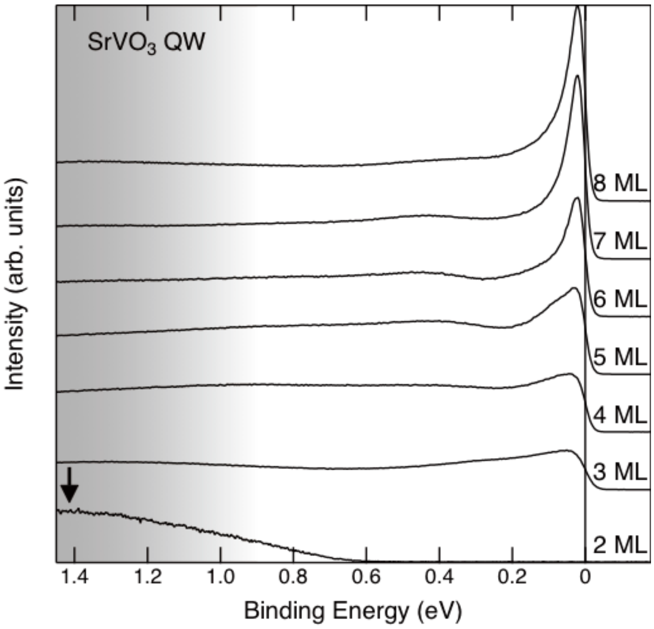


**Figure S3. Raw ARPES spectra at $k_F$ for $SrVO_3$ quantum-well structures in a wider energy range.** The arrow denotes the peak position of the incoherent part (lower Hubbard band), which is in good agreement with the previous AIPES results[17].

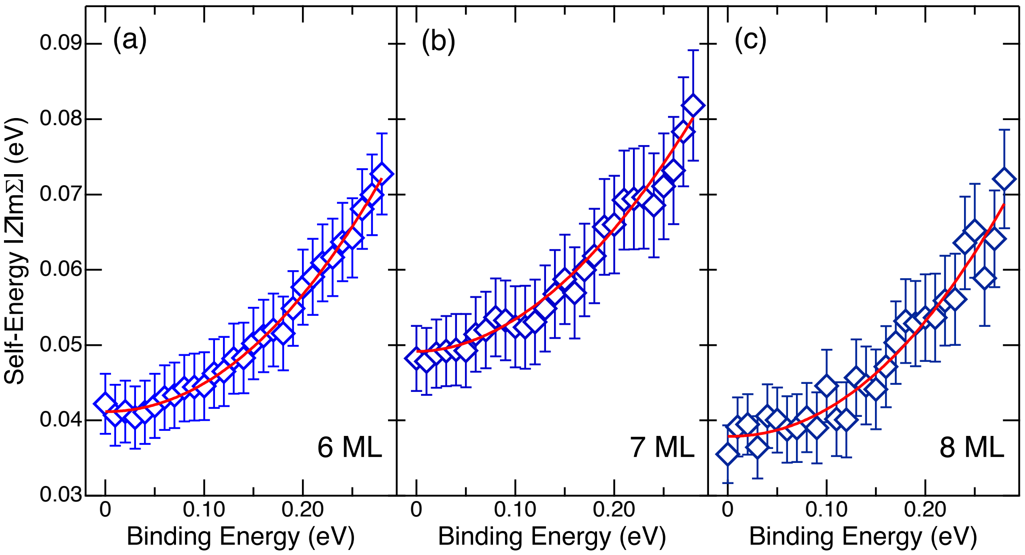


**Fig. S4 Self-energy for $SrVO_3$ QW states in the Fermi liquid regime.** (**a**)–(**c**) Imaginary part of self-energy $\mathrm{Im}\Sigma(\omega)$ for the $n = 1$ state of 6-, 7-, and 8-ML $SrVO_3$ QW structures. The solid curves are the result of parabola fitting (the fitting using Eq. (1) with $\alpha = 2$).

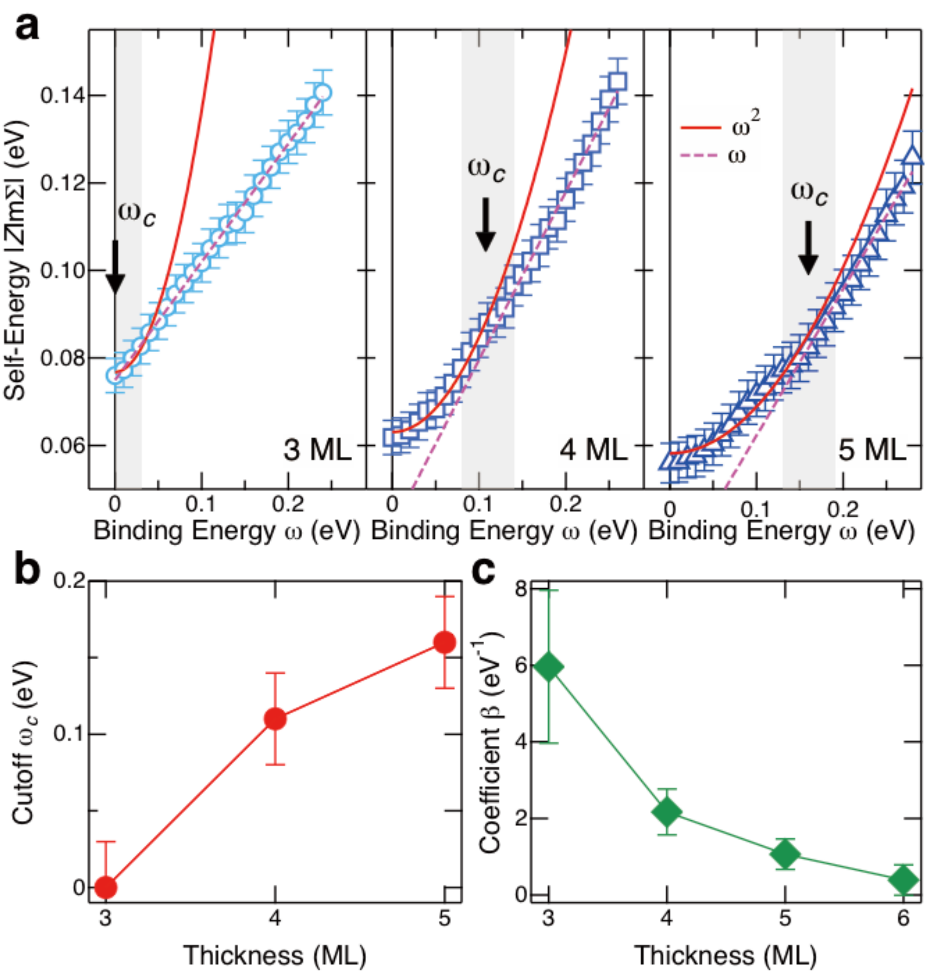


**Figure S5. Estimation of the Fermi-liquid cutoff energy in $SrVO_3$ quantum-well structures.** (**a**) The fitting results for experimentally obtained $\mathrm{Im}\Sigma(\omega)$ using Eq. (S3). (**b**), (**c**) Respectively, the plots of estimated $\omega_c$ and $\beta$ as a function layer thickness.
